# PROMOTING PROVIDER ADHERENCE TO THE MEDICATION M OF THE AGE-FRIENDLY 4MS FRAMEWORK

**DOI:** 10.1093/geroni/igad104.1861

**Published:** 2023-12-21

**Authors:** Ugochi Ohuabunwa, Leela Kantamneni

**Affiliations:** Emory University, Atlanta, Georgia, United States; Emory University, Atlanta, Georgia, United States

## Abstract

**BACKGROUND:**

The Age Friendly Health Systems (AFHS) initiative promotes use of the 4Ms Framework: We sought to evaluate provider adherence to protocols addressing the medication M of the framework.

**METHODS:**

The project was conducted among patients > 65years treated in the inpatient (IP), outpatient (OP) and emergency departments (ED) of Grady Memorial Hospital (GMH). We implemented 2 approaches to improve provider prescribing among older adults 1) Using a clinical decision support tool via the electronic health record Epic Systems®, a best practice advisory (BPA) offered individual provider feedback regarding potentially inappropriate medications (PIMs) ordered and offered safer alternative medications. 2)A pharmacist-led daily medication review with provider feedback on PIMs in the Acute Care for Elderly (ACE) Unit. Primary outcome: Percentage of PIMs discontinued. Secondary outcomes: Percentage of BPA vs pharmacist recommendations accepted, discharge PIMs, Length of Stay (LOS), mobility, delirium and Katz scores, 30-day readmission rate. RESULTS: 8270 BPA alerts fired for 3291 patients, mean age 73.7, with 5709 BPAs in IP, 1462 in ED & 1083 in OP settings. Providers responded to alerts by overriding BPA (10.2%), canceling BPA (60.4%), removing alerted PIM (20.6%), ordering alternative medication (3.5%).. With the pharmacist led intervention, ACE Unit providers accepted 47.2% recommendations, discontinued 17.9% PIMs . 19% of ACE and 1.1% of GMU patients were discharged without PIMs. 30-day readmission rate was 19.1% (ACE) vs 27% (GMU) (P = 0.31).

**Conclusion:**

There was a higher provider acceptance rate with the pharmacist led intervention but no significant difference in PIM discontinuation rate

